# Supplementary figures and images for: An open and continuously updated fern tree of life
Source: Front Plant Sci. 2022 Aug 24;13:909768. doi: 10.3389/fpls.2022.909768 (PMC9449725; doi:10.3389/fpls.2022.909768)

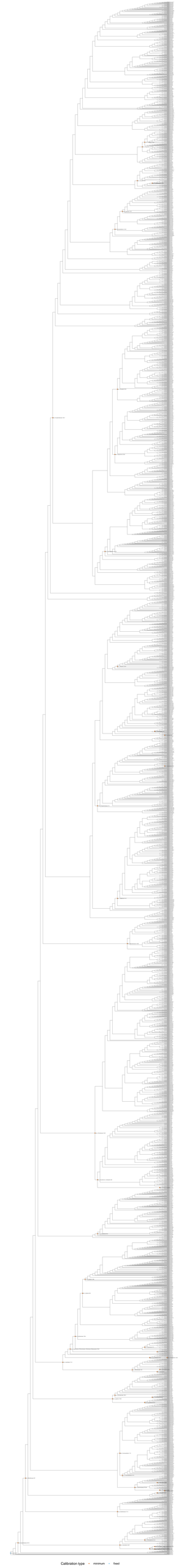

Supplement: Supplementary file 1 [file Data_Sheet_1.PDF]

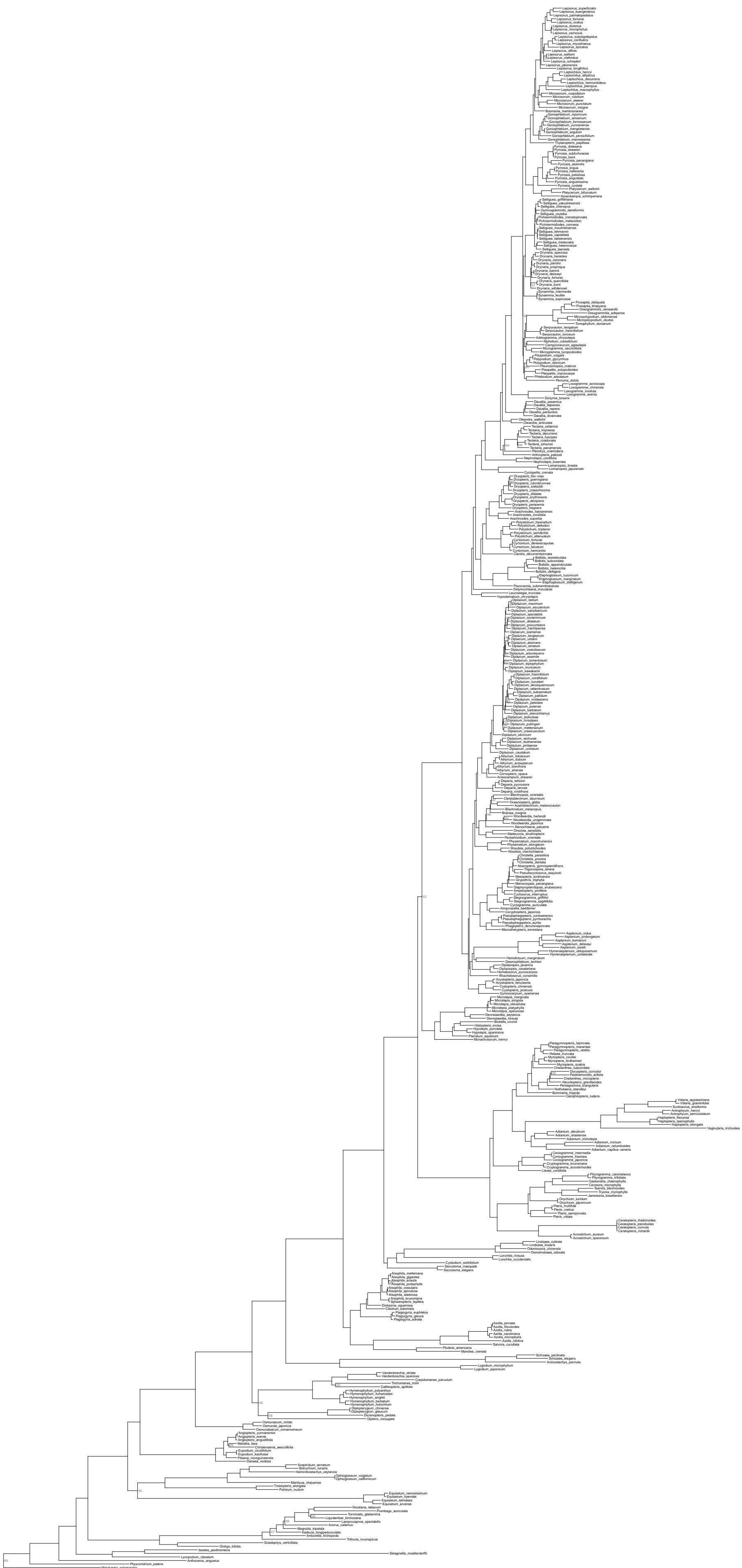

Supplement: Supplementary file 2 [file Data_Sheet_2.PDF]

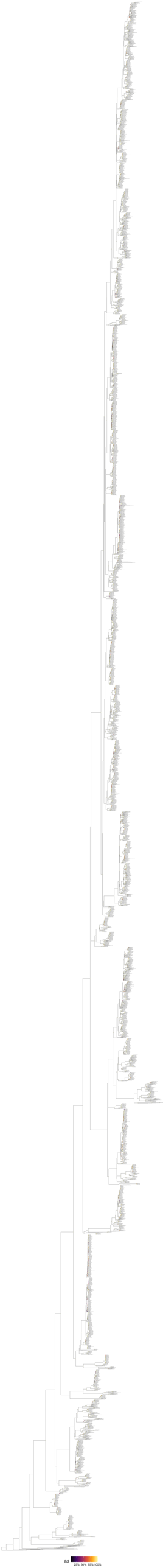

Supplement: Supplementary file 3 [file Data_Sheet_3.PDF]

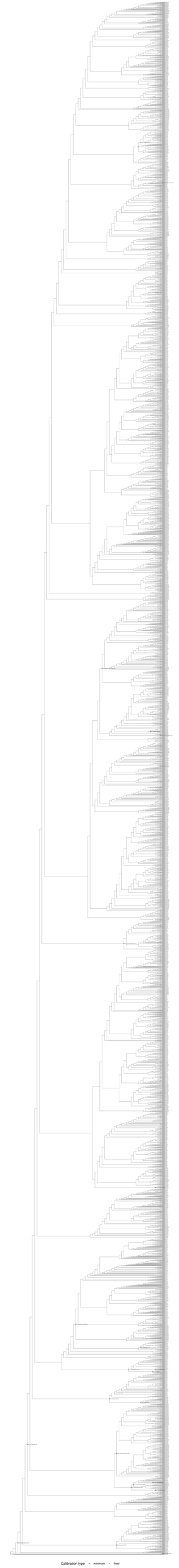

Supplement: Supplementary file 4 [file Data_Sheet_4.PDF]
