# Supplementary material for: An open and continuously updated fern tree of life
Source: Front Plant Sci. 2022 Aug 24;13:909768. doi: 10.3389/fpls.2022.909768 (PMC9449725; doi:10.3389/fpls.2022.909768)
Supplement: Supplementary file 10 [file Data_Sheet_10.pdf]

Appendix A1

Tables and Figures

Table S1. Differences in genera included as accepted in “pteridocat” taxonomic database v1.0.0 (https://github.com/fernphy/pteridocat) vs. PPGI (Pteridophyte Phylogeny Group I, 2016) (Table_S1.csv). “diff” is type of difference (pteridocat genus not in PPGI or PPGI genus not in pteridocat). “n_species” is the number of accepted species in each genus (pteridocat) or estimated number of species (PPGI). References in “note” column provided at end of this appendix. “World Ferns” refers to Hassler (2022), which was modified to create pteridocat.

Table S2. Fossils used as calibration points for molecular dating (Table_S2.csv). “n_fos” is the fossil ID number used in the “ferncal” database v1.0.1 (https://github.com/fernphy/ferncal). “minimum_age” is the age used for calibration (Ma); “lower” is the lower limit of the oldest stratigraphic age for the fossil (Ma); “upper” is the upper limit of the oldest stratigraphic age for the fossil (Ma). For detailed notes and full references for each fossil, see Appendix A2.

Table S3. GenBank accessions identified as rogues by all-by-all BLAST (Table_S3.csv). “q_family” is the family of the GenBank query (accession). “s_family” is the family of the top three best matches (only cases where the top three matches all belong to the same family included). All accessions in this table were excluded from further analysis.

Table S4. GenBank accessions used in this study (Table_S4.csv). “species” is the species name as it appears in the phylogenetic tree. “seq_len” is the total number of bases excluding missing bases (“?”, “N”, or “-”) and only includes bases belonging to loci used in this study (not the entire accession). “sci_name” is the scientific name in the pteridocat taxonomic database. “ncbi_name” is the scientific name used in the NCBI taxonomic database version 2022-05-01. “ncbi_taxid” is the NCBI taxonomy database unique identifier. “outgroup_status” is “FALSE” for ingroup taxa (ferns) and “TRUE” for outgroup taxa.

Method	n	%
Publication	138	2.67%
Manual	166	3.22%
Monophyletic	658	12.75%
Unjoined	1,383	26.80%
Voucher	2,816	54.56%
Total	5,161	

Table S5. Methods used to join accessions across loci for Sanger dataset. “manual” only used for Thelypteridaceae (see Methods). Count is number of species.

Taxonomic level	Monophyly	n	%
Order	Yes	11	100%
Suborder	Yes	6	100%
Family	Monotypic	4	8.3%
Family	Yes	44	91.7%
Subfamily	Monotypic	2	8.0%
Subfamily	Yes	22	88.0%
Subfamily	No	1	4.0%
Genus	Monotypic	67	20.1%
Genus	Yes	223	67.0%
Genus	No	43	12.9%

Table S6. Monophyly by taxonomic level for ferns in FTOL.

Table S7. Non-monophyletic fern genera in FTOL (Table_S7.csv). “number_tips” is the number of tips (species) in the genus. “delta_tips” is the number of tips that do not belong to the genus but are included in the clade descending from the most common recent ancestor of the genus. “number_intruders” is the number of intruding taxa into the genus. “number_outliers” is the number of tips (species) belonging to the genus that appear in other clades. Status of intruders and outliers was determined using the default cutoff level (0.5) in the R package “MonoPhy” (Schwery and O’Meara, 2016).

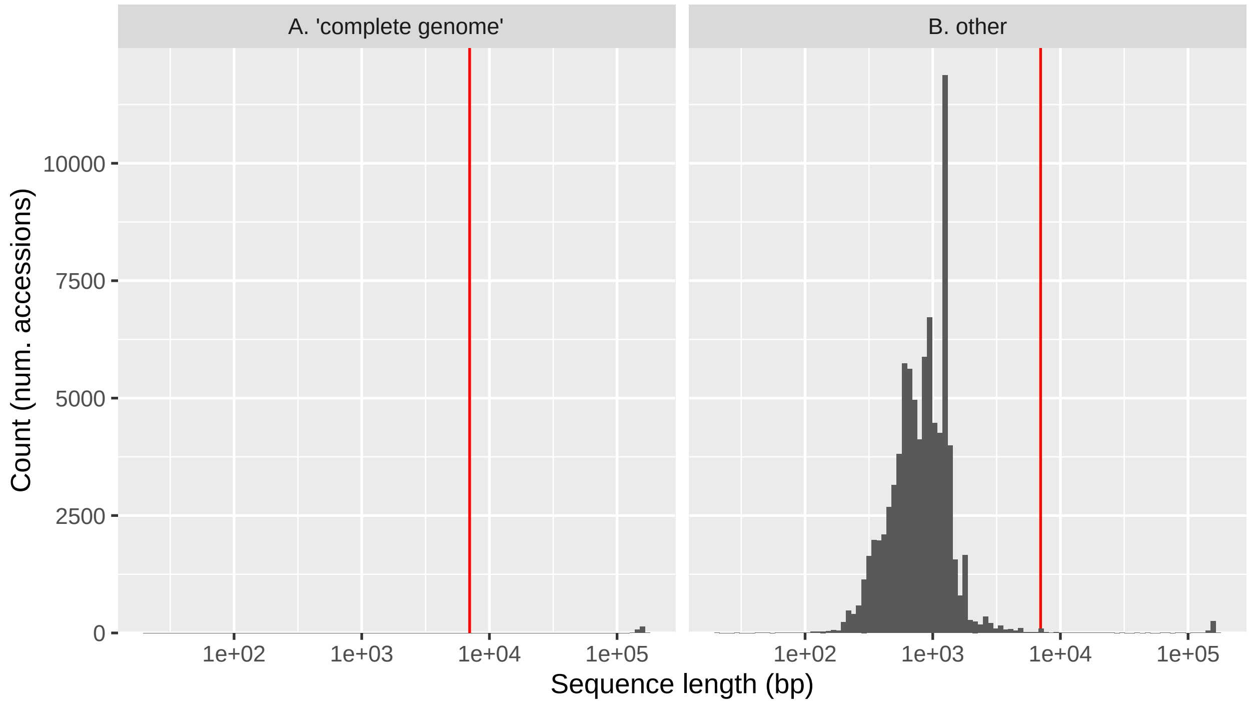

Figure S1. Histogram of sequence lengths (bp; log-scale) of fern accessions in GenBank release v249. Fern accessions obtained by querying GenBank for "Polypodiopsida[ORGN]" with length 10–200,000 bp. **A.** Sequences with the term 'complete genome' in their description (*e.g.*, "Angiopteris evecta chloroplast, complete genome"). **B.** Other sequences (those without 'complete genome' in their description). Vertical red line at 7,000 bp indicates empirical length cutoff used to differentiate between between Sanger (≤ 7,000 bp) and plastome (> 7,000 bp) accessions. Note that "other" includes some whole plastome accessions that cannot be distinguished based on description alone (e.g., "UNVERIFIED: Nephrolepis biserrata plastid sequence"); these are mostly above 7,000 bp.

Figure S2. Consensus phylogeny (Sanger dataset) with node labels indicating placement of fossil constraints (Figure_S2.pdf). Number for each constraint is age in Ma. Outgroup taxon *Zygnema circumcarinatum* (algae) pruned prior to dating analysis and not shown.

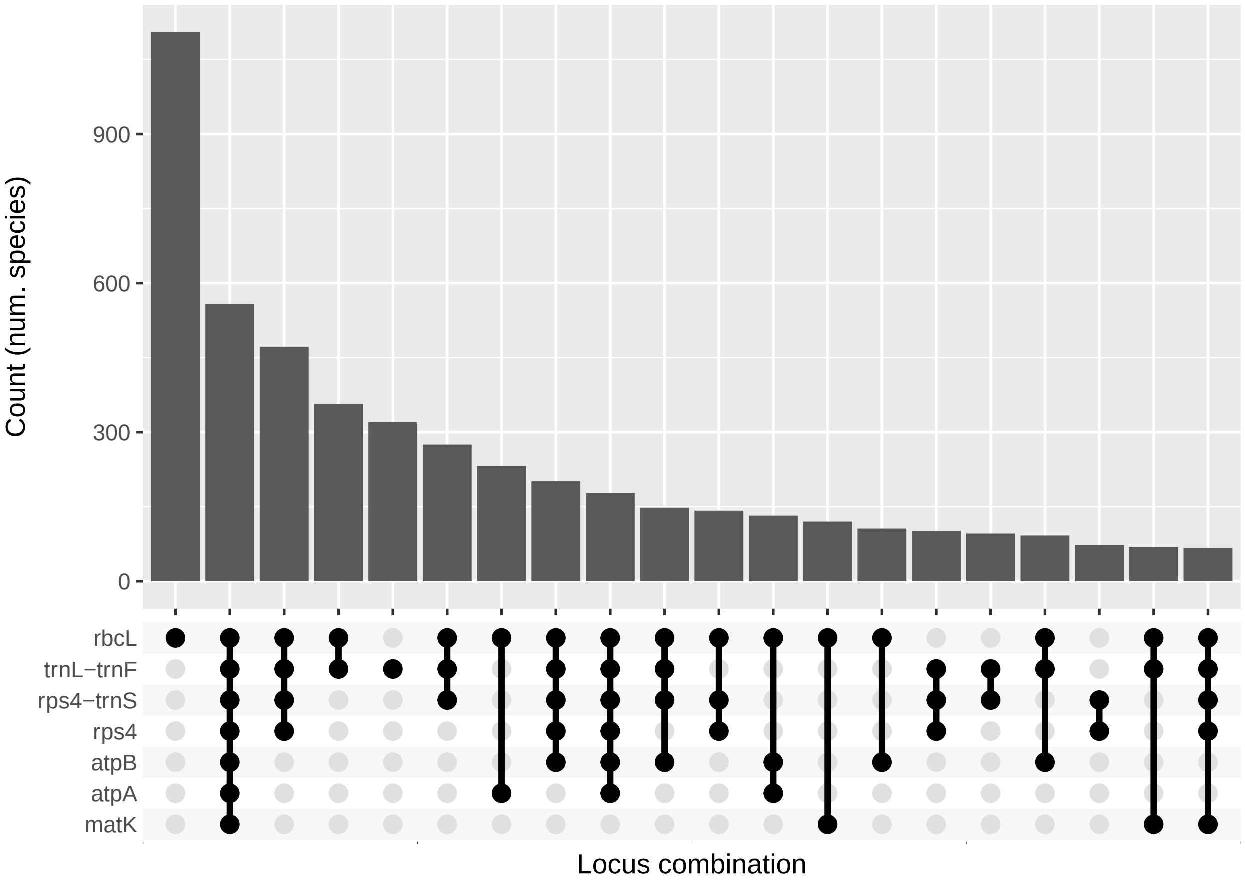

Figure S3. Frequency of locus combinations by species, Sanger dataset. Only top 20 combinations shown.

Figure S4. Consensus plastome (backbone) phylogeny (Figure_S4.pdf). Values at nodes are bootstrap support (%); only values less than 100% are shown. Outgroup taxon *Zygnema circumcarinatum* (algae) not shown.

Figure S5. Consensus phylogeny (Sanger dataset) with node labels showing bootstrap support (%) (Figure_S5.pdf). Darker colors indicate lower support. The backbone (plastome) phylogeny was used as a constraint tree, so all clades present in the backbone phylogeny tree receive 100% bootstrap support. Outgroup taxon *Zygnema circumcarinatum* (algae) not shown.

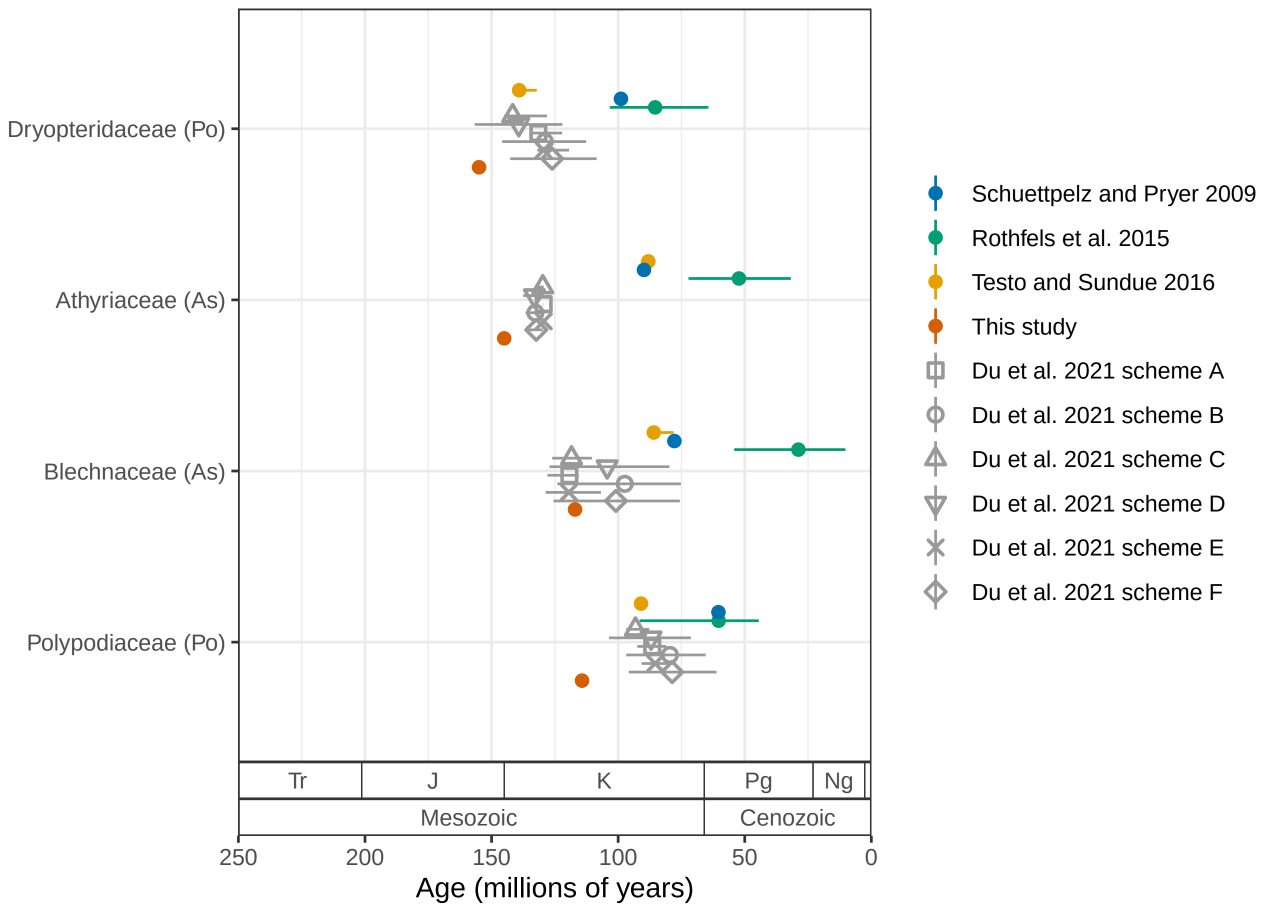

Figure S6. Stem age of fern families (Ma) estimated by selected studies, only including families with stem ages that were provided across all studies. For studies that used methods with confidence intervals, error bars indicate lower and upper 95% highest posterior density levels and point indicates median (Rothfels et al., 2015; Testo and Sundue, 2016; Du et al., 2021). For other studies, point indicates best (most likely) estimate (Schuettpelz and Pryer, 2009; this study). Du et al. (2021) used six different dating schemes; for details, see that paper. Codes in parentheses after family names indicate major clade as in Figure 3. Period name abbreviations as in Figure 5.

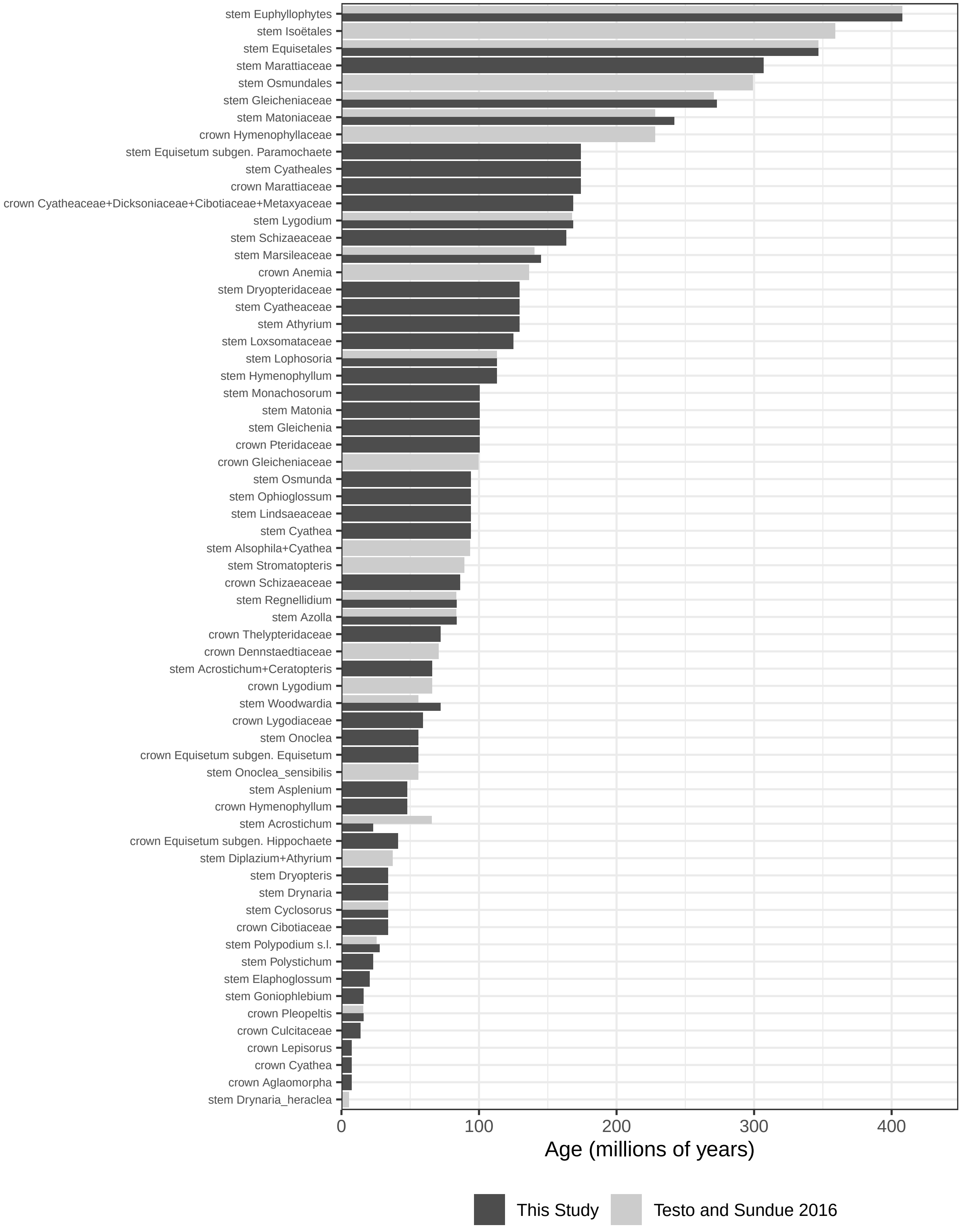

Figure S7. Age of nodes constrained by fossils in this study vs. Testo and Sundue (2016). Wide bars indicate the node was only constrained in one study; narrow bars indicate the the node was constrained in both studies.

Figure S8. Consensus phylogeny (Sanger dataset) with node labels indicating placement of fossil constraints of Testo and Sundue (2016) (Figure_S8.pdf). Number for each constraint is age in Ma. Outgroup taxon *Zygnema circumcarinatum* (algae) pruned prior to dating analysis and not shown.

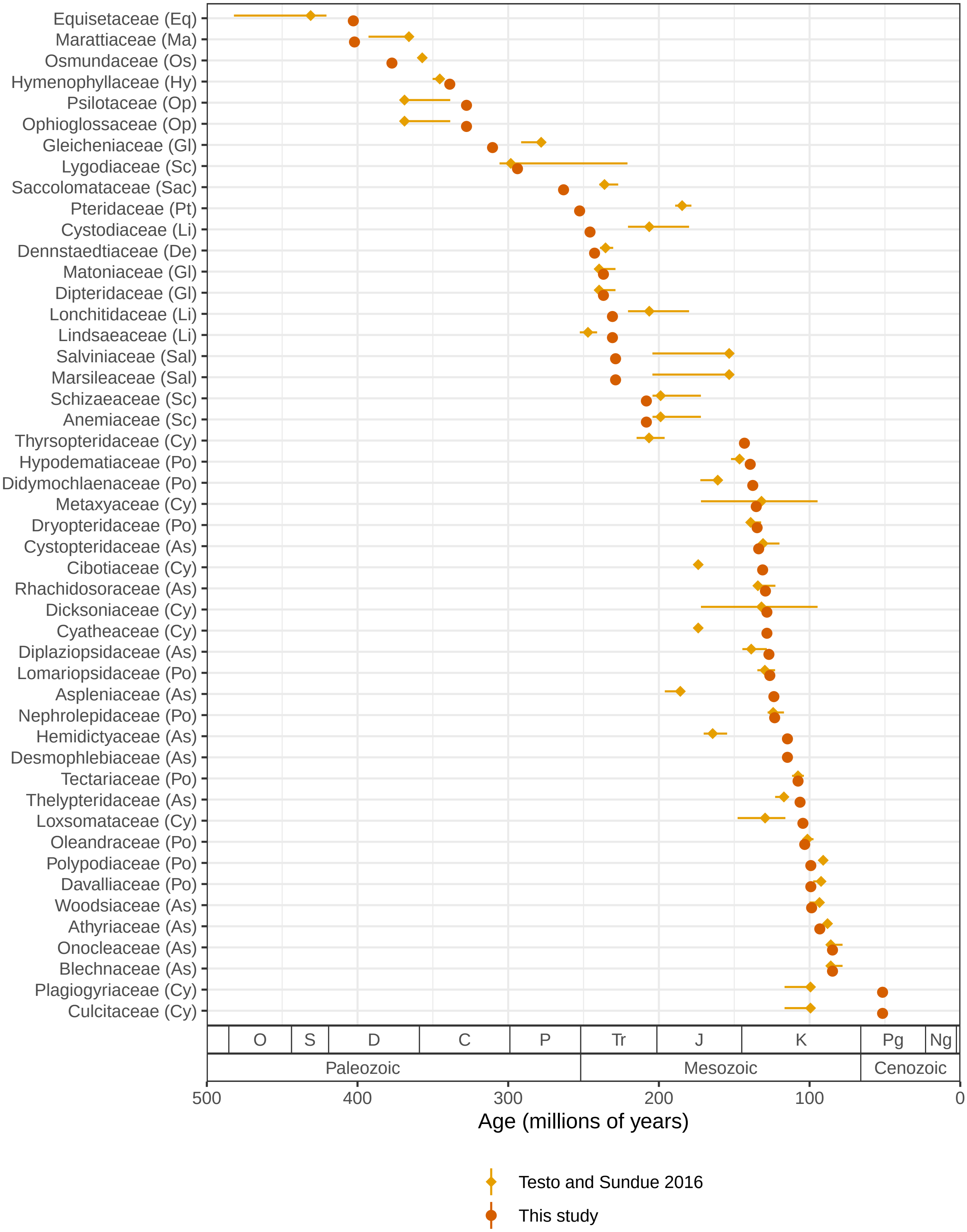

Figure S9. Comparison of stem age of fern families (Ma) estimated by Testo and Sundue (2016) and those estimated using methodology (treePL) and phylogeny (FTOL) of the current study with the fossil constraints of Testo and Sundue (2016). For Testo and Sundue (2016), error bars in-

dicate lower and upper 95% highest posterior density levels and point indicates median For this study, point indicates best (most likely) estimate. Codes in parentheses after family names indicate major clade as in Figure 3. Period name abbreviations as in Figure 5.

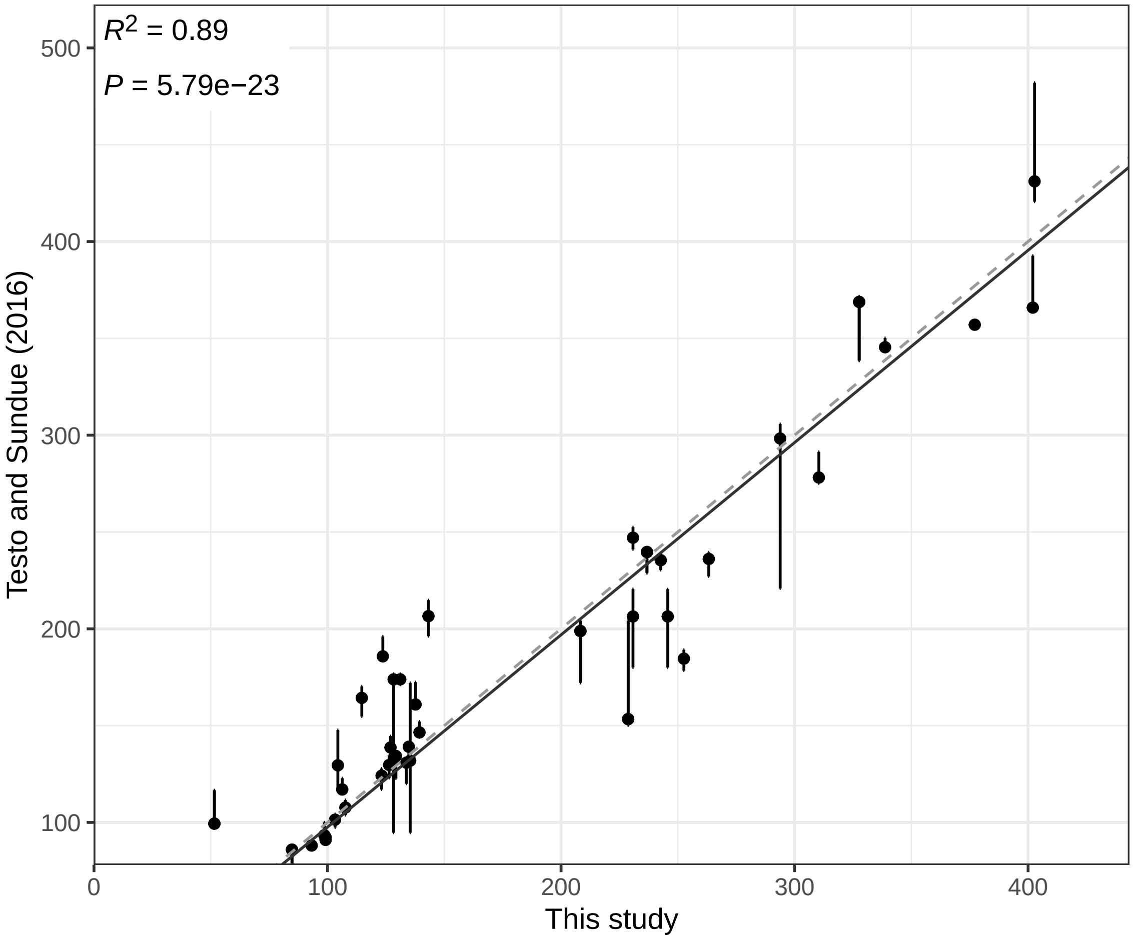

Figure S10. Comparison of stem age of fern families estimated by Testo and Sundue (2016) and those estimated using methodology (treePL) and phylogeny (FTOL) of the current study with the fossil constraints of Testo and Sundue (2016). All units in millions of years (Ma). For Testo and Sundue (2016), error bars indicate lower and upper 95% highest posterior density levels and point indicates median. For this study, point indicates best (most likely) estimate. Dashed indicates 1:1 relationship. Solid line indicates linear model fit.

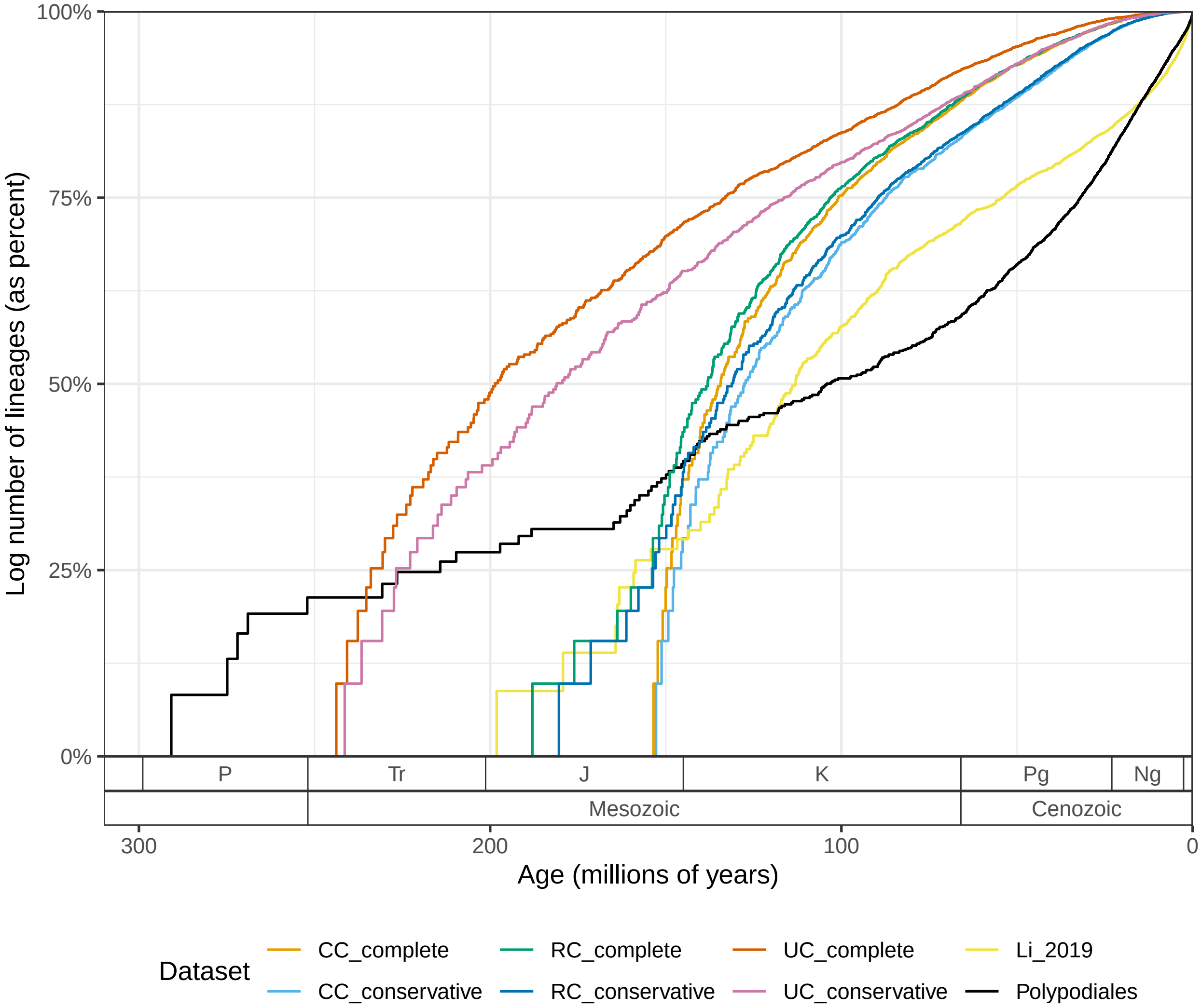

Figure S11. Lineage through time (LTT) plots for the fern order Polypodiales (black; this study) and angiosperms (colored lines; Li et al., 2019; Ramírez-Barahona et al., 2020). Angiosperm datasets labeled with "CC", "RC", or "UC" correspond to the six analysis schemes of Ramírez-Barahona et al. (2020); for details, see that paper. Log number of lineages shown as percent of total to enable comparison across trees.

References

Almeida, T. E., Salino, A., Dubuisson, J.-Y., and Hennequin, S. (2017). *Adetogramma* (Polypodiaceae), a new monotypic fern genus segregated from Polypodium. *PhytoKeys* 78, 109–131. doi:10.3897/phytokeys.78.12189.

Applequist, W. L. (2019). Report of the Nomenclature Committee for Vascular Plants: 70. *Taxon* 68, 847–854. doi:10.1002/tax.12113.

Chen, C.-W., Sundue, M. A., Kuo, L.-Y., Teng, W.-C., and Huang, Y.-M. (2017). Phylogenetic analyses place the monotypic *Dryopolystichum* within Lomariopsidaceae. *PhytoKeys* 78, 83–107. doi:10.3897/phytokeys.78.12040.

Dong, S.-Y., Chen, C.-W., Tan, S.-S., Zhao, H.-G., Zuo, Z.-Y., Chao, Y.-S., et al. (2018). New insights on the phylogeny of *Tectaria* (Tectariaceae), with special reference to *Polydictyum* as a distinct lineage: New insights on the phylogeny of *Tectaria*. *Journal of Systematics and Evolution* 56, 139–147. doi:10.1111/jse.12292.

Dong, S.-Y., and Zuo, Z.-Y. (2018). On the recognition of *Gymnosphaera* as a distinct genus in Cyatheaceae. *Annals of the Missouri Botanical Garden* 103, 1–23. doi:10.3417/2017049.

Dong, S., Zuo, Z., Xiao, Y., Huang, L., Pham, V. T., Phan, K. L., et al. (2020). A contribution to *Gymnosphaera* (Cyatheaceae) in mainland Asia: Two new species, reinstatement of *Cyathea bonii* , and their phylogenetic positions. *Journal of Systematics and Evolution*, jse.12679. doi:10.1111/jse.12679.

Du, X., Lu, J., Zhang, L., Wen, J., Kuo, L., Mynssen, C. M., et al. (2021). Simultaneous diversification of Polypodiales and angiosperms in the Mesozoic. *Cladistics* 37, 518–539. doi:10.1111/cla.12457.

Fawcett, S., and Smith, A. (2021). *A Generic Classification of the Thelypteridaceae*. Botanical Research Institute of Texas Press.

Fraser-Jenkins, C. R., Gandhi, K. N., and Kholia, B. S. (2016). *An annotated checklist of Indian Pteridophytes*. Dehra Dun (India): Bishen Singh Mahendra Pal Singh.

George, L. O., Pryer, K. M., Kao, T.-T., Huiet, L., and Windham, M. D. (2019). *Baja*: A new monospecific genus segregated from *Cheilanthes* s.l. (Pteridaceae). *Systematic Botany* 44, 471–482. doi:10.1600/036364419x15620113920536.

Hassler, M. (2022). World Ferns. Synonymic Checklist and Distribution of Ferns and Lycophytes of the World. Available at: www.worldplants.de/ferns/ [Accessed January 1, 2022].

Li, H.-T., Yi, T.-S., Gao, L.-M., Ma, P.-F., Zhang, T., Yang, J.-B., et al. (2019). Origin of angiosperms and the puzzle of the Jurassic gap. *Nature Plants* 5, 461–470. doi:10.1038/s41477-019-0421-0.

Liu, H., Rakotondrainibe, F., Hennequin, S., and Schneider, H. (2020a). The significance of *Rouxopteris* (Gleicheniaceae, Polypodiopsida): A new genus endemic to the Madagascan region. *Plant Systematics and Evolution* 306, 30. doi:10.1007/s00606-020-01657-9.

Liu, H., Schuettpelz, E., and Schneider, H. (2020b). Evaluating the status of fern and lycophyte nothotaxa in the context of the Pteridophyte Phylogeny Group classification (PPG I). *Journal of Systematics and Evolution* 58, 988–1002. doi:10.1111/jse.12641.

Lu, N. T., Zhou, X., Zhang, L., Knapp, R., Li, C., Fan, X., et al. (2019). A global plastid phylogeny of the cliff fern family Woodsiaceae and a two-genus classification of Woodsiaceae with the description of × *Woodsimatium* nothogen. nov. *Taxon* 68, 1149–1172. doi:10.1002/tax.12180.

Moran, R. C., Garrison Hanks, J., and Sundue, M. (2019). Phylogenetic relationships of Neotropical lady ferns (Athyriaceae), with a description of *Ephemeropteris*, gen. nov. *TAXON* 68, 425–441. doi:10.1002/tax.12063.

Parris, B. S., and Sundue, M. A. (2020). The fern genus *Oreogrammitis* (Grammitidoideae: Polypodiaceae) re-defined. *Phytotaxa* 436, 45–54. doi:10.11646/phytotaxa.436.1.4.

Ponce, M. M., and Scataglini, M. A. (2022). Phylogenetic position of South American *Cheilanthes* (Cheilanthoideae, Pteridaceae): Advances in the generic circumscription and segregation of the new genus *Mineirella*. *Journal of Systematics and Evolution* 60, 266–280. doi:10.1111/jse.12723.

Pteridophyte Phylogeny Group I (2016). A community-derived classification for extant lycophytes and ferns. *Journal of Systematics and Evolution* 54, 563–603. doi:10.1111/jse.12229.

Ramírez-Barahona, S., Sauquet, H., and Magallón, S. (2020). The delayed and geographically heterogeneous diversification of flowering plant families. *Nature Ecology & Evolution* 4, 1232–1238. doi:gg45rb.

Rothfels, C. J., Li, F.-W., Sigel, E. M., Huiet, L., Larsson, A., Burge, D. O., et al. (2015). The evolutionary history of ferns inferred from 25 low-copy nuclear genes. *American Journal of Botany* 102, 1–19. doi:10.3732/ajb.1500089.

Schneider, H., Kreier, H.-P., Hovenkamp, P., and Janssen, T. (2008). Phylogenetic relationships of the fern genus *Christiopteris* shed new light onto the classification and biogeography of drynarioid ferns. *Botanical Journal of the Linnean Society* 157, 645–656. doi:10.1111/j.1095-8339.2008.00825.x.

Schuettpelz, E., and Pryer, K. M. (2009). Evidence for a Cenozoic radiation of ferns in an angiosperm-dominated canopy. *Proceedings of the National Academy of Sciences of the United States of America* 106, 11200–11205. doi:10.1073/pnas.0811136106.

Schwery, O., and O'Meara, B. C. (2016). MonoPhy: a simple R package to find and visualize monophyly issues. *PeerJ Computer Science* 2, e56. doi:10.7717/peerj-cs.56.

Shalisko, V., Sundue, M. A., Villalobos-Arámbula, A. R., Muñiz-Castro, M. Á., and Vázquez-García, J. A. (2019). Taxonomic novelties in grammitid ferns (Polypodiaceae) from the Neotropics and Madagascar supported by molecular data. *Phytotaxa* 394, 176. doi:10.11646/phytotaxa.394.3.1.

Shang, H., Sundue, M., Wei, R., Wei, X.-P., Luo, J.-J., Liu, L., et al. (2018). *Hiya*: A new genus segregated from *Hypolepis* in the fern family Dennstaedtiaceae, based on phylogenetic evidence and character evolution. *Molecular Phylogenetics and Evolution*. doi:10.1016/j.ympev.2018.04.038.

Testo, W. L., Field, A. R., Sessa, E. B., Sundue, M. A., and Hall, B. (2019). Phylogenetic and morphological analyses support the resurrection of *Dendroconche* and the recognition of two new genera in Polypodiaceae subfamily Microsoroideae. 44, 1–16. doi:10.1600/036364419X15650157948607.

Testo, W., and Sundue, M. (2016). A 4000-species dataset provides new insight into the evolution of ferns. *Molecular Phylogenetics and Evolution* 105, 200–211. doi:10.1016/j.ympev.2016.09.003.

Wei, R., Ebihara, A., Zhu, Y. M., Zhao, C. F., Hennequin, S., and Zhang, X. C. (2018). A total-evidence phylogeny of the lady fern genus *Athyrium* Roth (Athyriaceae) with a new infrageneric classification. *Molecular Phylogenetics and Evolution* 119, 25–36. doi:10.1016/j.ympev.2017.10.019.

Wilson, K. L. (2016). Report of the General Committee: 15. *Taxon* 65, 1150–1151. doi:10.12705/655.14.

Zhang, L., Fan, X., Petchsri, S., Zhou, L., Pollawatn, R., Zhang, X., et al. (2020). Evolutionary relationships of the ancient fern lineage the adder's tongues (Ophioglossaceae) with description of *Sahashia* gen. nov. *Cladistics* 36, 380–393. doi:10.1111/cla.12408.

Zhang, L., Zhou, X. M., Lu, N. T., and Zhang, L. B. (2017). Phylogeny of the fern subfamily Pteridoideae (Pteridaceae; Pteridophyta), with the description of a new genus: *Gastoniella*. *Molecular Phylogenetics and Evolution* 109, 59–72. doi:10.1016/j.ympev.2016.12.037.

Zhao, C. F., Wei, R., Zhang, X. C., and Xiang, Q. P. (2019). Backbone phylogeny of *Lepisorus* (Polypodiaceae) and a novel infrageneric classification based on the total evidence from plastid and morphological data. *Cladistics*, 1–24. doi:10.1111/cla.12403.

Zhou, X.-M., Zhang, L., Chen, C.-W., Li, C.-X., Huang, Y.-M., Chen, D.-K., et al. (2017). A plastid phylogeny and character evolution of the Old World fern genus *Pyrrosia* (Polypodiaceae) with

the description of a new genus: *Hovenkampia* (Polypodiaceae). *Molecular Phylogenetics and Evolution* 114, 271–294. doi:10.1016/j.ympev.2017.06.020.
